# Predictors of Postoperative Physical Therapy Utilization After Common Orthopaedic Procedures

**DOI:** 10.2106/JBJS.OA.26.00192

**Published:** 2026-07-24

**Authors:** Neel Bhardwaj, Sahil Dadoo, Rachit Saggar, Derrick Lin, Siya Bhardwaj, Jonathan D. Hughes

**Affiliations:** 1 University of Pittsburgh School of Medicine, Pittsburgh, Pennsylvania; 2 Department of Orthopaedic Surgery, University of Pittsburgh Medical Center, Pittsburgh, Pennsylvania; 3 David Geffen School of Medicine at UCLA, Los Angeles, California; 4 Department of Psychology, University of California, Los Angeles, California

## Abstract

**Background:**

Physical therapy (PT) is essential to recovery after orthopaedic surgery, yet predictors of postoperative PT utilization across procedure types remain poorly understood. This study aimed to identify demographic and clinical predictors of PT utilization after 8 common orthopaedic procedures using a diverse national cohort.

**Methods:**

Patients undergoing 8 orthopaedic procedures were identified from the National Institutes of Health All of Us Research Program. The primary outcome was PT utilization within 180 days of surgery. Sequential multivariable logistic regression evaluated predictors of PT utilization, adjusting for demographics, comorbidities, insurance, employment, preoperative PT, 0- to 14-day postoperative opioid prescriptions, and procedure type. Procedure-stratified analyses were performed. Statistical significance was set as p < 0.050.

**Results:**

Among 12,666 patients (59% female; mean age, 58 ± 13 years), including 1,531 (12%) Hispanic/Latino and 1,445 (11%) Black patients, overall postoperative PT utilization was 44%. Female sex was associated with lower odds of PT utilization compared with male sex (adjusted odds ratio [aOR], 0.85; 95% confidence interval [CI], 0.78 to 0.92; p < 0.001), an effect that persisted after full adjustment. Preoperative PT (aOR, 8.91; 95% CI, 7.75-10.23; p < 0.001) and 0- to 14-day postoperative opioid prescriptions (aOR, 1.66; 95% CI, 1.50-1.83; p < 0.001) were independently associated with higher PT utilization. The association between female sex and lower PT utilization reached statistical significance in the total knee arthroplasty subgroup only in procedure-stratified analyses (aOR, 0.78; 95% CI, 0.67-0.91; p = 0.001). PT utilization was associated with lower 90-day emergency department visits (aOR, 0.67; 95% CI, 0.59-0.76; p < 0.001).

**Conclusions:**

Preoperative PT and early postoperative opioid prescriptions were the strongest predictors of postoperative PT utilization. Female sex was associated with lower PT utilization after comprehensive adjustment, although structural and unmeasured factors may partially account for this finding. These findings identify factors associated with rehabilitation access after orthopaedic surgery. Preoperative PT engagement, equitable opioid-prescribing practices, and strategies to address insurance-related barriers warrant further investigation as potential approaches to improve postoperative rehabilitation access.

**Level of Evidence:**

Level III, Prognostic. See Instructions for Authors for a complete description of levels of evidence.

## Introduction

Physical therapy (PT) is a cornerstone of recovery after orthopaedic surgery, providing functional restoration, complication prevention, and long-term outcomes^1-4^. Despite this, PT utilization rates are highly variable, with reported PT engagement rates ranging from 30% to 80% depending on the procedure and patient population^5,6^.

Sex-based differences in orthopaedic care have received increasing attention, prompting calls for improved sex-stratified reporting^7^. Female patients are less likely to be referred for arthroplasty, present at later disease stages, and experience inferior functional outcomes^8-12^. Preoperative PT has been associated with improved function and quality of life after total joint arthroplasty, suggesting that preoperative rehabilitation may influence postoperative recovery; however, whether this reflects or drives subsequent utilization remains unclear^13^.

The primary aim of this study was to identify demographic and clinical predictors of postoperative PT utilization after 8 common orthopaedic procedures using a diverse national cohort from the National Institutes of Health (NIH) All of Us Research Program. It was hypothesized that preoperative PT and postoperative opioid prescriptions would be independently associated with higher postoperative PT utilization. It was further hypothesized that female sex would be associated with lower PT utilization based on documented sex-based disparities in orthopaedic care delivery and that this association would be partially explained by differences in insurance coverage and employment status^8-12^.

## Methods

### Data Source

This study used deidentified data from the NIH All of Us Research Program Registered Tier Curated Data Repository (CDR), a longitudinal cohort of over 800,000 participants with linked electronic health record (EHR) data^14,15^.

### Study Population

Adult patients (18 years or older) who underwent 1 of 8 common orthopaedic procedures were identified using Current Procedural Terminology (CPT) codes: total knee arthroplasty (TKA; 27447), arthroscopic meniscectomy (29881, 29880), arthroscopic meniscus repair (29882), anterior cruciate ligament reconstruction (29888), total hip arthroplasty (27130), arthroscopic rotator cuff repair (29827), arthroscopic biceps tenodesis (29828), and total shoulder arthroplasty (23472). The above 8 procedures were selected as the highest volume arthroscopic, reconstructive, and arthroplasty procedures within the NIH All of Us CDR, ensuring adequate sample size for procedure-stratified analyses. For patients with multiple qualifying procedures, only the earliest procedure was retained to eliminate within-patient correlation.

### Predictor Variables

Predictor variables included age, sex, Hispanic/Latino ethnicity, and Black race. Other racial categories (Asian [n = 107], multiracial [n = 485], and other/unspecified [n = 371]) had insufficient sample sizes for stable multivariable modeling and are presented descriptively in Supplementary Table I. Comorbidities (diabetes, hypertension, obesity, depression, anxiety, hyperlipidemia, chronic kidney disease, cardiovascular disease, pulmonary disease, osteoarthritis, and chronic pain) were identified from condition_occurrence records on or before the index procedure. Insurance (Medicare, Medicaid, private, military/VA, uninsured, other, and unknown) and employment status (employed vs. not employed) were derived from survey data. Geographic location was not available in this CDR release, precluding adjustment for region. Additional variables included preoperative PT, defined as any PT-related CPT code within 180 days before surgery, and postoperative opioid prescriptions, identified from the “drug exposure” table within 0 to 14 days of surgery by matching drug concept names for oxycodone, hydrocodone, tramadol, morphine, codeine, fentanyl, hydromorphone, oxymorphone, and tapentadol. The drug exposure table captures prescribed medications rather than confirmed pharmacy dispensing or patient consumption. The 0- to 14-day opioid window was selected to reflect the acute postoperative prescribing period consistent with Centers for Disease Control (CDC) opioid-prescribing recommendations and to reduce temporal overlap with the PT outcome window^16^.

### Outcome Measures

The primary outcome was any PT-related CPT code within 180 days of index surgery. A secondary PT outcome, “adequate PT utilization,” was defined as ≥6 PT visits within 90 days of surgery. Clinical outcomes included 90-day emergency department (ED) visits, 2-year revision surgery, 30-day readmission, and 90–180-day persistent opioid prescribing.

### Statistical Analysis

Baseline characteristics were compared using Mann-Whitney *U* and chi-square tests. A sequential multivariable logistic regression approach was used to evaluate predictors of PT utilization: a base model adjusting for demographics, comorbidities, and procedure type; additional adjustment for depression and anxiety; additional adjustment for preoperative PT; additional adjustment for postoperative opioid prescriptions; and a fully adjusted model including all covariates. E-value analysis quantified robustness to unmeasured confounding^17^. A capture-bias sensitivity analysis was performed in patients with high EHR engagement (≥2 preoperative and ≥3 postoperative encounters at contributing health systems). Random-effects meta-analysis (DerSimonian-Laird) pooled procedure-stratified estimates and quantified between-procedure heterogeneity (*I*^2^, Cochran Q). Statistical significance was set at p < 0.050. Causal mediation analysis was performed to evaluate whether 0- to 14-day postoperative opioid prescriptions mediated the association between Hispanic ethnicity and PT utilization, using counterfactual mediation decomposition with 1,000 bootstrap resamples. This analysis assumes no unmeasured mediator-outcome confounding and correct temporal ordering, supported by the 0- to 14-day opioid window preceding most PT initiation.

## Results

### Study Population

**Fig. 1:**
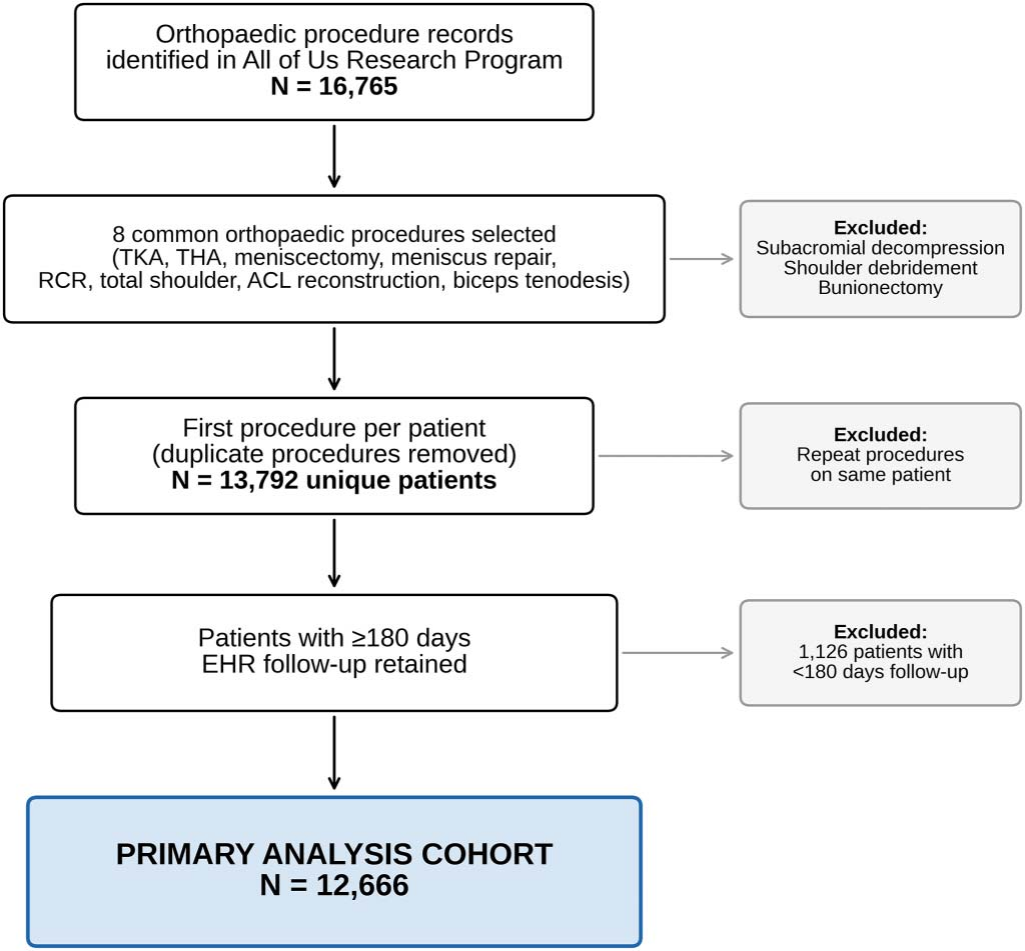
Study flow diagram. Flow diagram depicting the identification and selection of the study cohort from the NIH All of Us Research Program Registered Tier Curated Data Repository. A total of 16,765 orthopaedic procedure records were screened. After selection of 8 common orthopaedic procedures, restriction to the first procedure per patient, and exclusion of patients with fewer than 180 days of EHR follow-up, 12,666 unique patients were included in the primary analysis cohort. EHR = electronic health record, N = number of patients, NIH = National Institutes of Health, THA = total hip arthroplasty, and TKA = total knee arthroplasty.

Of 16,765 orthopaedic procedure records initially screened, 12,666 patients were included in the primary analysis cohort (Fig. 1 and Table I). The mean age of the total cohort was 58 ± 13 years, 7,427 (59%) patients were female, 1,531 (12%) patients were Hispanic/Latino, and 1,445 (11%) patients were Black. Female patients had higher rates of obesity, depression, anxiety, and chronic pain and were more likely to be Hispanic and Black (all p < 0.001; Table I).

**TABLE I:** Patient Characteristics by Sex (N = 12,666)

Characteristics	Female (n = 7,427)	Male (n = 5,239)	p
Demographics			
Age, yr, mean ± SD	58 ± 13	58 ± 13	0.452
Hispanic/Latino	980 (13)	551 (11)	**<0.001**
Black race	970 (13)	475 (9)	**<0.001**
Comorbidities			
Diabetes mellitus	1,134 (15)	847 (16)	0.178
Hypertension	2,714 (37)	2,053 (39)	**0.003**
Obesity	2,071 (28)	1,038 (20)	**<0.001**
Depression	1,701 (23)	774 (15)	**<0.001**
Anxiety	1,594 (22)	696 (13)	**<0.001**
Chronic pain	3,149 (42)	1,797 (34)	**<0.001**
Procedure type			**<0.001**
TKA	2,508 (34)	1,479 (28)	
Arthroscopic meniscectomy	2,191 (30)	1,638 (31)	
THA	1,236 (17)	963 (18)	
Arthroscopic rotator cuff repair	753 (10)	653 (12)	
Total shoulder arthroplasty	392 (5)	304 (6)	
ACL reconstruction	208 (3)	98 (2)	
Biceps tenodesis	46 (1)	71 (1)	
Meniscus repair	93 (1)	33 (1)	
Preoperative care			
Preoperative PT (180 d)	963 (13)	707 (14)	0.401
Postoperative course			
Opioid prescription (0-14 d)	5,199 (70)	3,898 (74)	**<0.001**
Postoperative PT (180 d)	3,155 (43)	2,459 (47)	**<0.001**
30-d inpatient readmission	938 (13)	578 (11)	**0.007**
90-d emergency department visit	727 (10)	572 (11)	**0.042**
90-d postoperative complication	164 (2)	127 (2)	0.460

ACL = anterior cruciate ligament, PT = physical therapy, THA = total hip arthroplasty, and TKA = total knee arthroplasty.

Values in bold indicate p < 0.050.

### PT Utilization

Overall PT utilization within 180 days after surgery was 44%, ranging from 27% (arthroscopic meniscectomy) to 62% (TKA). In the fully adjusted model (Table II), preoperative PT demonstrated the strongest association with PT utilization (adjusted odds ratio [aOR], 8.91; 95% confidence interval [CI], 7.75-10.23; p < 0.001), followed by military/VA insurance compared with private insurance (aOR, 3.58; 95% CI, 2.90-4.41; p < 0.001) and 0- to 14-day postoperative opioid prescriptions (aOR, 1.66; 95% CI, 1.50-1.83; p < 0.001). Female sex was independently associated with lower PT utilization (aOR, 0.85; 95% CI, 0.78-0.92; p < 0.001). Hispanic ethnicity was not significantly associated with PT utilization after full adjustment (aOR, 0.93; 95% CI, 0.82-1.06; p = 0.301). Chronic pain (aOR, 0.70; 95% CI, 0.64-0.78; p < 0.001), unemployment (aOR, 0.83; 95% CI, 0.76-0.92; p < 0.001), and Medicaid insurance (aOR, 0.87; 95% CI, 0.76-1.00; p = 0.046) were independently associated with lower PT utilization.

**TABLE II:** Fully Adjusted Multivariable Model: Predictors of Postoperative Physical Therapy Utilization (N = 12,666)

Variables	aOR	95% CI	p
Sociodemographic			
Female sex (vs. male)	**0.85**	0.78-0.92	**<0.001**
Age (per yr)	1.00	0.99-1.00	0.082
Hispanic/Latino ethnicity (vs. non-Hispanic)	0.93	0.82-1.06	0.301
Black race (vs. non-Black)	0.94	0.83-1.07	0.356
Comorbidities			
Obesity	**1.18**	1.06-1.32	**0.002**
Diabetes mellitus	0.95	0.84-1.08	0.436
Hypertension	**0.89**	0.79-0.99	**0.034**
Depression	0.94	0.82-1.07	0.334
Anxiety	**1.20**	1.06-1.36	**0.005**
Chronic pain	**0.70**	0.64-0.78	**<0.001**
Insurance type (vs. Private)			
Medicare	0.98	0.87-1.10	0.714
Medicaid	**0.87**	0.76-1.00	**0.046**
Military/VA	**3.58**	2.90-4.41	**<0.001**
Uninsured	**0.62**	0.48-0.81	**<0.001**
Other	1.05	0.82-1.35	0.682
Unknown	**0.78**	0.68-0.90	**<0.001**
Employment status			
Not employed (vs. employed)	**0.83**	0.76-0.92	**<0.001**
Perioperative care pathway			
Preoperative PT	**8.91**	7.75-10.23	**<0.001**
Postoperative opioid Rx (0-14 d)	**1.66**	1.50-1.83	**<0.001**
Procedure type (vs. TKA)			
THA	**0.68**	0.60-0.78	**<0.001**
Arthroscopic rotator cuff repair	**0.51**	0.44-0.60	**<0.001**
Total shoulder arthroplasty	**0.74**	0.60-0.91	**0.004**
Arthroscopic meniscectomy	**0.24**	0.21-0.27	**<0.001**
ACL reconstruction	0.91	0.68-1.22	0.534
Meniscus repair	**0.58**	0.40-0.84	**0.004**
Biceps tenodesis	**0.32**	0.20-0.51	**<0.001**

Model adjusted for age, sex, Hispanic ethnicity, Black race, comorbidities (diabetes, hypertension, obesity, depression, anxiety, chronic pain, hyperlipidemia, chronic kidney disease, cardiovascular disease, pulmonary disease, and osteoarthritis), insurance type, employment status, preoperative PT, 0- to 14-day postoperative opioid prescriptions, and procedure type. TKA served as the reference category for procedure type. Values in bold indicate p < 0.050. ACL = anterior cruciate ligament, aOR = adjusted odds ratio, CI = confidence interval, PT = physical therapy, Rx = prescription, TKA = total knee arthroplasty, and VA = Veterans Affairs.

### Procedure-Stratified Analysis

**Fig. 2:**
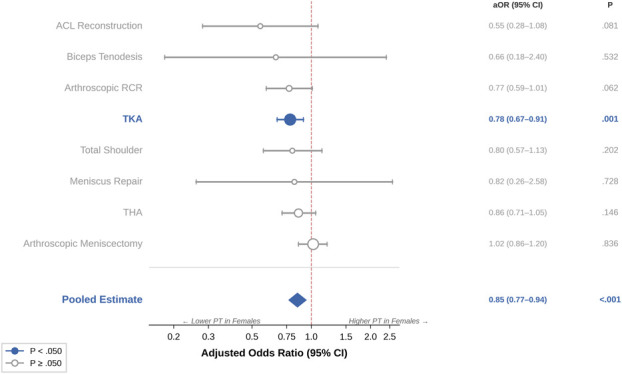
Procedure-stratified adjusted odds of physical therapy utilization for female versus male patients. Forest plot depicting the aOR and 95% CI for PT utilization within 180 days of surgery among female patients compared with male patients, stratified by procedure type. Estimates were derived from procedure-specific logistic regression models using the fully adjusted model including age, Hispanic/Latino ethnicity, Black race, comorbidities, insurance type, employment status, preoperative PT, and 0- to 14-day postoperative opioid prescriptions. Procedures are ordered by effect size. Filled circles indicate p < 0.050; open circles indicate p ≥ 0.050. The diamond represents the pooled estimate from random-effects meta-analysis (aOR = 0.85; 95% CI, 0.77-0.94; I^2^ = 14%). ACL = anterior cruciate ligament, aOR = adjusted odds ratio, CI = confidence interval, PT = physical therapy, RCR = rotator cuff repair, THA = total hip arthroplasty, and TKA = total knee arthroplasty.

In procedure-stratified analyses using the fully adjusted model (Supplementary Table II), the sex-based disparity in PT utilization was statistically significant only after TKA (aOR, 0.78; 95% CI, 0.67-0.91; p = 0.001). Random-effects meta-analysis (DerSimonian-Laird) yielded a pooled female sex (aOR, 0.85; 95% CI, 0.77-0.94) with low between-procedure heterogeneity (I^2^ = 14%; Q = 8.15; p = 0.32; Fig. 2).

### Preoperative PT and Opioid Prescribing

**Fig. 3:**
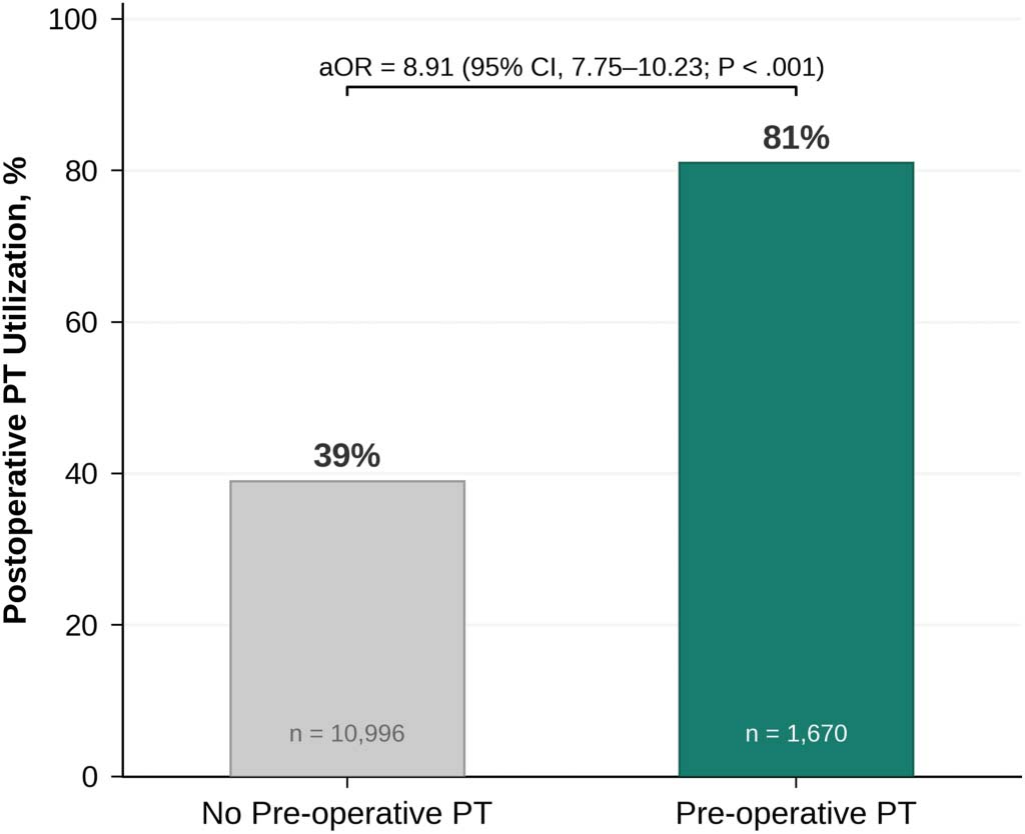
Postoperative physical therapy utilization by preoperative PT status. Postoperative PT utilization within 180 days of surgery among patients who did (n = 1,670; 81%) and did not (n = 10,996; 39%) receive preoperative PT (p < 0.001, χ^2^ test). The aOR was derived from the fully adjusted logistic regression model (aOR = 8.91; 95% CI, 7.75-10.23; p < 0.001). aOR = adjusted odds ratio, CI = confidence interval, and PT = physical therapy.

Overall, 1,670 (13%) patients received preoperative PT within 180 days before surgery. Patients with preoperative PT had markedly higher postoperative utilization than those without (81% vs. 39%; p < 0.001; Fig. 3).

In addition, 0- to 14-day opioid prescriptions were documented for 72% of patients. Hispanic patients were substantially less likely to receive opioid prescriptions than non-Hispanic patients (52% vs. 75%; p < 0.001).

### Sequential Modeling

Sequential adjustment demonstrated distinct patterns for the sex and ethnicity associations (Supplementary Fig. 1; Supplementary Table III). The female sex association remained stable through adjustment for depression, anxiety, preoperative PT, and opioid prescriptions (aOR range, 0.74-0.76) but weakened in the fully adjusted model including insurance and employment (aOR, 0.85; 95% CI, 0.78-0.92; p < 0.001).

By contrast, the association between Hispanic ethnicity and lower PT utilization progressively weakened as covariates were added: base model (aOR, 0.83; p = 0.002), weakening with preoperative PT (aOR, 0.86; p = 0.021), and opioid prescriptions (aOR, 0.92; p = 0.216, to aOR, 0.93; p = 0.301) in the fully adjusted model. In formal mediation analysis, the indirect effect of Hispanic ethnicity on PT utilization through opioid prescriptions was −0.89 percentage points (95% CI, −1.21 to −0.62), indicating that lower opioid-prescribing rates among Hispanic patients accounted for a statistically significant reduction in PT utilization. The direct effect not operating through opioid prescriptions was not significant (−1.28 percentage points; 95% CI, −3.71 to +1.18).

### PT Utilization and Clinical Outcomes

In secondary analyses, PT utilization was associated with lower 90-day ED visits (aOR, 0.67; 95% CI, 0.59-0.76; p < 0.001). By contrast, PT utilization was associated with higher 30-day readmission (aOR, 1.56; 95% CI, 1.38-1.75; p < 0.001) and persistent opioid prescribing at 90 to 180 days (aOR, 1.38; 95% CI, 1.26-1.52; p < 0.001). PT utilization was not significantly associated with 2-year revision surgery (aOR, 0.72; 95% CI, 0.51-1.02; p = 0.063), although the overall revision rate was low (1.3%). Because PT utilization was measured over 180 days and could have been initiated after outcomes occurring at 30 or 90 days, these associations do not establish temporal precedence of PT relative to the outcome.

### Adequate PT Utilization

Overall, 2,487 (19.7%) met the adequate PT threshold (≥6 visits within 90 days). In the fully adjusted model, the female sex association was weaker at this threshold (aOR, 0.90; 95% CI, 0.81-1.00; p = 0.044) compared with any PT utilization (aOR, 0.85), with TKA the only procedure reaching significance (aOR, 0.79; 95% CI, 0.66-0.94; p = 0.007).

### Sensitivity Analyses

In a capture-bias sensitivity analysis of 9,865 patients (78%) with high EHR engagement, female sex was associated with lower PT utilization (aOR, 0.71; 95% CI, 0.65-0.78; p < 0.001), a larger effect than in the full cohort (aOR, 0.85). If undercapture preferentially affected female patients, restricting to a higher capture subset would move the estimate toward the null; the opposite finding suggests that the sex disparity is not an artifact of differential capture. E-value analysis indicated that the preoperative PT association (E-value, 5.42) was highly robust to unmeasured confounding, while the female sex association (E-value, 1.39) was more susceptible given the smaller effect size.

## Discussion

The most important finding of this study was that preoperative PT and early postoperative opioid prescriptions were the strongest predictors of postoperative PT utilization after common orthopaedic procedures. Insurance type and employment status were also independently associated with PT utilization. Female sex was associated with modestly lower PT utilization after adjustment for these factors, with the association statistically significant only after TKA in procedure-stratified analyses.

Preoperative PT was the strongest predictor of postoperative utilization, far exceeding any demographic, comorbidity, or pharmacologic factor. This likely reflects established care pathways (scheduling infrastructure, provider relationships, and insurance preauthorization) and patient-level factors, including failure to attend initial evaluation, health literacy, and access to care^18-20^. These findings align with prior prehabilitation evidence^13^.

Postoperative opioid prescriptions demonstrated the second strongest association with PT utilization. The association between Hispanic ethnicity and lower PT utilization was no longer significant after adjustment for opioid prescriptions, suggesting that previously described disparities in opioid prescribing among Hispanic patients may partially explain the observed difference in PT utilization^16,21-24^. Opioid prescriptions may also reflect surgical complexity or pain severity not directly measured in this cohort^25,26^. Formal mediation analysis showed a statistically significant indirect effect of Hispanic ethnicity on PT utilization through opioid prescriptions, whereas the direct effect was not significant. This finding should be interpreted with caution because the analysis assumes no unmeasured confounding between opioid prescribing and PT utilization. Addressing equity in opioid prescribing may nonetheless have implications for rehabilitation access among Hispanic patients^27^.

Female sex was associated with modestly lower PT utilization, although this association weakened substantially after adjustment for insurance and employment. The weakening of the sex effect after insurance and employment adjustment suggests that structural factors, rather than intrinsic sex differences, may account for much of the observed disparity. Unmeasured factors such as caregiving responsibilities could also contribute^28^. The association between female sex and lower PT utilization reached statistical significance in the TKA subgroup only, although low between-procedure heterogeneity (I^2^ = 14%) and consistent point estimates across procedures suggest that this reflects the larger TKA sample size rather than a procedure-specific effect. TKA and other arthroplasty procedures require more structured rehabilitation than arthroscopic meniscectomy^29,30^. At the adequate-PT threshold, the female sex association was weaker (aOR, 0.90 vs. 0.85 for any PT), suggesting that the difference is driven more by PT initiation than by engagement once treatment begins.

Insurance and employment were independently associated with PT utilization, consistent with prior evidence^31^; military/VA insurance was associated with higher utilization^32^ and Medicaid with lower utilization. Chronic pain was associated with lower PT utilization, a finding that may reflect fear of movement (kinesiophobia) and concern that physical activity will exacerbate symptoms, leading to avoidance behavior^33^. The “unknown” insurance category was associated with lower PT utilization (aOR, 0.78; p < 0.001), suggesting that missing insurance data were not random; this group may include patients with fragmented coverage, recent coverage transitions, or incomplete survey response, any of which could independently limit rehabilitation access.

PT was associated with lower ED visits but higher readmission and persistent opioid prescribing. This pattern likely reflects confounding by indication, as patients with complications or persistent pain are both more likely to receive PT referrals and more likely to require readmission or continued opioid therapy. PT utilization was also measured over 180 days and may have been initiated after outcomes occurring at 30 or 90 days, limiting directional inference. These associations should be considered hypothesis generating.

This study has several limitations. First, PT utilization was ascertained from EHR-captured CPT codes, which may underestimate true engagement if patients received PT outside contributing healthcare systems; while this undercapture likely biases toward the null, differential capture across demographic groups cannot be excluded. The any-PT outcome does not capture rehabilitation intensity or adherence, although the adequate-PT analysis partially addresses this limitation. Second, opioid prescriptions were ascertained from the drug exposure table, which captures prescribed medications rather than confirmed dispensing. Chronic pain was ascertained from ICD-10 codes in the condition_occurrence table, which is an imprecise proxy for the clinical construct and may reflect documentation patterns rather than validated pain severity. Third, the sequential modeling approach cannot establish causal pathways, and the female sex E-value (1.39) suggests susceptibility to unmeasured confounding. Plausible unmeasured confounders such as caregiving burden, transportation access, work schedule flexibility, and surgeon referral patterns could approach or exceed this threshold.

## Conclusion

Preoperative PT and early postoperative opioid prescriptions were the strongest predictors of postoperative PT utilization. Female sex was associated with lower PT utilization after comprehensive adjustment, although structural and unmeasured factors may partially account for this finding. These findings identify factors associated with rehabilitation access after orthopaedic surgery. Preoperative PT engagement, equitable opioid-prescribing practices, and strategies to address insurance-related barriers warrant further investigation as potential approaches to improve postoperative rehabilitation access.

## Appendix

Supporting material provided by the authors is posted with the online version of this article as a data supplement at jbjs.org (http://links.lww.com/JBJSOA/B289).
